# Genetically Depauperate and Still Successful: Few Multilocus Genotypes of the Introduced Parthenogenetic Weevil *Naupactus cervinus* (Coleoptera: Curculionidae) Prevail in the Continental United States

**DOI:** 10.3390/insects14020113

**Published:** 2023-01-22

**Authors:** Marcela S. Rodriguero, Viviana A. Confalonieri, Ava Mackay Smith, Mary Kate Dornon, Eleanor Zagoren, Alice Palmer, Andrea S. Sequeira

**Affiliations:** 1 Departamento de Ecología, Genética y Evolución, Facultad de Ciencias Exactas y Naturales, Universidad de Buenos Aires, Ciudad Autónoma de Buenos Aires 1428, Argentina; 2 Instituto de Ecología, Genética y Evolución (IEGEBA), CONICET–Universidad de Buenos Aires, Ciudad Autónoma de Buenos Aires 1428, Argentina; 3 Department of Biological Sciences, Wellesley College, Wellesley, MA 02481, USA

**Keywords:** agricultural pests, polyphagy, prevalent invader genotype, general purpose genotype

## Abstract

**Simple Summary:**

The Fuller’s rose weevil *Naupactus cervinus* has become a globally invasive pest of several ornamental plants and fruit trees. This weevil has caused severe economic losses, and eggs laid on fruit are a quarantine barrier for several countries’ exports of fruit to markets in East Asia. Previous studies revealed that a genotype with high colonization ability successfully expanded throughout most continents, even in areas of inadequate environmental conditions, where this insect performs unexpectedly well, and that parthenogenesis may have helped to expand its geographic range. Pre-existing variation might have been eroded either by natural selection, leading to fixation of this variant able to cope with different environmental conditions to those in the native range, or by repeated bottlenecks during the process of invasion. To better understand the ecology of this invasive pest, we enlarged the sampling in areas of introduction, such as the southern United States, and surveyed genetic variation through mitochondrial and nuclear sequences in 13 localities across three states. Our results indicate that the invader genotype, already identified, has also colonized the continental United States (US), supporting the hypothesis of a general-purpose genotype capable of coping with adverse conditions and enlarging its geographical range. Parthenogenesis, and its associated lack of recombination, may help in maintaining a general-purpose genotype that facilitates the colonization of distant, unsuitable areas. However, demographic advantages linked to parthenogenesis as the sole mode of reproduction are also possible.

**Abstract:**

*Naupactus cervinus* is a parthenogenetic weevil native to South America that is currently distributed worldwide. This flightless species is polyphagous and capable of modifying gene expression regimes for responding to stressful situations. *Naupactus cervinus* was first reported in the continental United States in 1879 and has rapidly colonized most of the world since. Previous studies suggested that an invader genotype successfully established even in areas of unsuitable environmental conditions. In the present work, we analyze mitochondrial and nuclear sequences from 71 individuals collected in 13 localities across three states in the southern US, in order to describe the genetic diversity in this area of introduction that has not yet been previously studied. Our results suggest that 97% of the samples carry the most prevalent invader genotype already reported, while the rest shows a close mitochondrial derivative. This would support the hypothesis of a general purpose genotype, with parthenogenesis and its associated lack of recombination maintaining the linkage of genetic variants capable of coping with adverse conditions and enlarging its geographical range. However, demographic advantages related to parthenogenetic reproduction as the main driver of geographic expansion (such as the foundation of a population with a single virgin female) cannot be ruled out. Given the historical introduction records and the prevalence of the invader genotype, it is possible that the continental US may act as a secondary source of introductions to other areas. We propose that both the parthenogenesis and scarce genetic variation in places of introduction may, in fact, be an asset that allows *N. cervinus* to thrive across a range of environmental conditions.

## 1. Introduction

Biological invasions are one of the most important drivers of the ongoing biodiversity crisis [1]. Invasive alien species represent an increasing threat to ecosystems: they have eroded biogeographic realms [2], have been identified as major drivers of native species extinction [3], caused regime shifts in recipient environments [4], caused degradation of ecosystem functions and services [5], and disrupted phylogenetic and functional diversity [6,7]. Additionally, an often-underrated consequence of invasions is the enormity of their socioeconomic impacts, such as the emergence and dissemination of infectious diseases [8,9] and economic cost at the hundreds of billions USD scale worldwide [10]. Insects are among the most invasive taxa and can have strong impacts on forest and crop productions and on domestic animals and human health. Particularly, the invasion of phytophagous insects through human activities, strongly related to rising globalization, such as commercial trade, leads to the establishment of new agricultural pests [11].

Curculionoidea is the largest and most diverse lineage of Metazoa [12], with several economically important forestry and agricultural pests, such as the boll weevil *Anthonomus grandis* Boheman, the fruit weevil *Naupactus xanthographus* (Germar), the coffee berry borer *Hypothenemus hampei* (Ferrari), the banded pine weevil *Pissodes castaneus* (De Geer), the eucalyptus snout beetle *Gonipterus platensis* Morelli, and the Fuller’s rose weevil *Naupactus cervinus* Boheman [13,14,15,16,17,18,19,20].

*Naupactus cervinus* is a highly polyphagous species from the tribe Naupactini [21,22] that causes severe damage to various economically important species, such as fruit trees [21] and other crop species, including citrus crop roots [13,23]. Native to South America, a series of events during the Ice Age shaped the genetic variation of this weevil, leading to two ecotypes with parapatric distribution, namely forest and grassland clades [19,24], with the first one ranging in the southernmost tip of the Atlantic Forest and the second one expanding into the Argentine Plains. Upon secondary contact of such divergent groups, hybrid genotypes have originated [25]. This apterous weevil has successfully established invasive populations in many countries via commercial trade well beyond its native range, including the US and Australia [19]. While the species reproduces via obligate parthenogenesis [26], sexually reproducing populations were believed to have existed until approximately 70 years ago [25,27,28].

In addition to an increased reproductive rate, parthenogenesis would be advantageous for the colonization of new environments by preventing the breakup of successful gene combinations [19,20]. Within the tribe Naupactini, several species have been found to reproduce parthenogenetically; interestingly, only flightless species have been found to employ this mode of reproduction [29]. Reduced flight capacity has been hypothesized to be related to parthenogenetic species colonization events in heterogeneous landscapes [30]. Thus, parthenogenesis may contribute to the enhanced colonization ability of insects in many ways.

Despite the fact that parthenogenesis usually reduces the amount of available genetic variation present in populations, established populations of *N. cervinus* within the native range harbor substantial variability [19,24] that may be explained by the history of past sexual reproduction [25] and diversification after acquisition of parthenogenetic reproduction a long time ago [24]. In contrast, populations within the introduced range are not expected to harbor significant levels of genetic variation, given that males have never been detected in any area of the introduced range and that introductions are recent, most probably following bottlenecks. However, explorations of the transcriptional plasticity of introduced populations of *N. cervinus* in the US and the native range have revealed intriguing patterns of host-specific expression and modulated responses to plant defenses [31]. For example, significantly different quantities of immune defense, detoxification, and host detection genes were found to be up-regulated in legume-feeding weevils, when compared to those feeding on other hosts. Even in the absence of genetic variation, parthenogenetic species can still become successful invaders, establishing themselves in novel areas, such as *N. cervinus*, which was first reported in California in 1879 and has since invaded at least 30 other states [32], as well as other countries worldwide.

The most probable ancestral area of *N. cervinus* is the Paranaense forest, where this weevil shows the highest genetic diversity and the broadest geographic distribution [19]. Through ecological niche modeling analysis, it was demonstrated that a few multi-locus genotypes of this parthenogenetic weevil (mostly “B–VII”, and its derivative “B–V”) successfully invaded areas that were modeled as having low to null probability of establishment [19]. Even though many multilocus genotypes were found in the native area surrounding the main port used for commercial trade, only a single clone and its derivatives were able to successfully establish in these distant locations, most probably through multiple independent introductions [20]. Therefore, these clones could be considered as members of an invasive lineage of *N. cervinus*, with pre-existing adaptations that would allow for a wide habitat tolerance [20], a hypothesis that deserves to be tested.

This contribution broadens the geographic scope of previous studies [19,20] by adding multiple localities from two distant areas within the continental US, namely from southeastern states (Georgia and Florida) and from the West coast (three counties in California), where it was presumably introduced around the 1870s [32,33]. In searching for evidence of genetic variation, we ask if *N. cervinus* populations in these areas harbor any genetic variation and which multi-locus genotypes are the most prevalent.

The main objective is to test the hypothesis that the one successful invader lineage found worldwide will also be identified as the most prevalent (and, therefore, successful) across the continental US. Indeed, if this were the case, this might support the notion that this successful lineage can thrive in a diversity of habitats and feed on a variety of taxing host plants, possibly due to pre-existing adaptations [20] and/or due to plasticity in gene expression variation related to the exploitation of a variety of food sources [31].

## 2. Materials and Methods

### 2.1. Sampling

**Figure 1:**
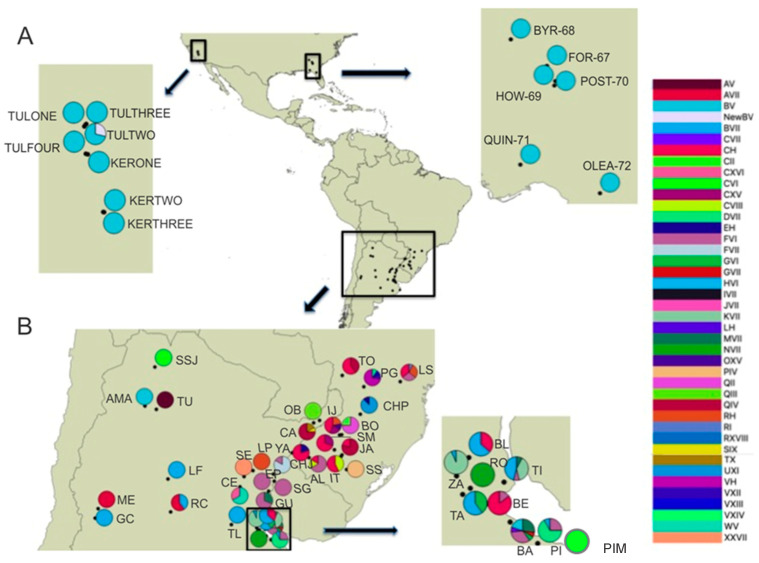
Geospatial distribution of combined genotypes of *Naupactus cervinus* for the Continental US (**A**) and the native range (**B**). Legend to the right lists all multilocus genotypes; pies indicate the relative presence of each multilocus genotype in each locality. Acronyms used for multilocus genotypes follow the nomenclature used by [19]. Localities are labeled according to codes in Table 1.

*Naupactus cervinus* specimens for this study were collected by placing a beating sheet under potential host plants. Specimens were placed in vials containing 100% ethanol until being processed for DNA studies. The 71 newly included specimens originated from 13 not previously sampled localities from two distantly separated areas (one in the East Coast and one on the West Coast), within the introduced range in the continental US. On the West Coast, samples originate from Tulare and Kern Co. in the central valley in California (spanning 150 km^2^), while those from the East coast originate from multiple counties in northern Florida and southern Georgia (spanning 450 km^2^) (Table 1; Figure 1A). Even though this sampling does not include every US state where historical records of *N. cervinus* have been recorded, it provides significant coverage of the longitudinal range of the current distribution within the continental US. Additionally, two native localities were added (AMA and SSJ, as in Table 1, Figure 1B), effectively extending the sampling of the native range in Argentina northwards towards the border with Bolivia in the Yungas Montana jungle, as well as a third one (PIM as in Table 1; Figure 1B) from the surrounding area of the most important commercial port in Buenos Aires Province.

### 2.2. DNA Extraction

Genomic DNA was extracted from the ethanol-preserved whole body tissue using the Qiagen DNeasy Blood and Tissue Kit (Qiagen, Inc., Valencia, CA, USA), following the manufacturer’s protocol for tissue samples. 

### 2.3. DNA Amplification and Sequencing

A segment of ca. 700 bp of the Cytochrome c Oxidase I (COI) gene was amplified using the specific primers S1718 and A2442 [36]. Additionally, a nuclear region of ca. 1100 bp that includes the region 3’ of the 18S rDNA gene, plus the complete ITS1 region (Internal Transcribed Spacer 1) and the 5’ region of the 5.8S rDNA gene, was amplified using the primers rDNA2 [37] and rDNA1.5.8S [38]. Polymerase chain reaction amplification and Sanger sequencing were carried out following [34].

We sequenced a total of 56 *N. cervinus* specimens for COI and 70 for ITS1 from the introduced area and 7 specimens for each gene from the native area. Alignment was performed using CLUSTAL W [39] and adjusted by eye. To check for the presence of pseudogenes, mitochondrial COI partial gene sequences were translated into aminoacid sequences using the invertebrate mitochondrial code with the program MEGA v. 5 [40]. Sequences obtained for both genes were aligned and compared to those obtained in previous works by [19,24,34,35] (Table 1 and Table S1). In this way, multilocus genotypes (i.e., COI-ITS1 variants) were identified according to the nomenclature already used by these authors (Table 1, Figure 1), yielding 59 combined genotypes for US. This sequence data was combined with a larger dataset, totaling 30 localities from the introduced range and 40 from the native range [19], the latter including the new seven samples reported in the present study (1 from AMA, 1 from SSJ, 1 from PIM, and 4 from BA).

### 2.4. Data Analysis

Estimates of genetic variation between samples within the introduced range and within the native range were calculated using DNAsp v.6 [41]. Statistics reported in Table 2 include the number of polymorphic sites, the average number of nucleotide differences *k* (aka Theta K) [42], and the average number of nucleotide differences per site between two sequences *Pi* [43,44] (equations 10.5 or 10.6 in [44]). Kst nucleotide-based statistics [−45] (eq. 10 in [45]) were calculated between seven geographically close locality groups in the introduced range (as indicated in Table 1), each one presumed to be the result of a single introduction event.

Statistical parsimony analysis was conducted with all individual COI and ITS1 sequences using the program TCS v. 1.21 [46] to generate haplotype and allele networks, respectively. The connection limit, excluding homoplastic changes, was set to 95%.

## 3. Results

**Figure 2:**
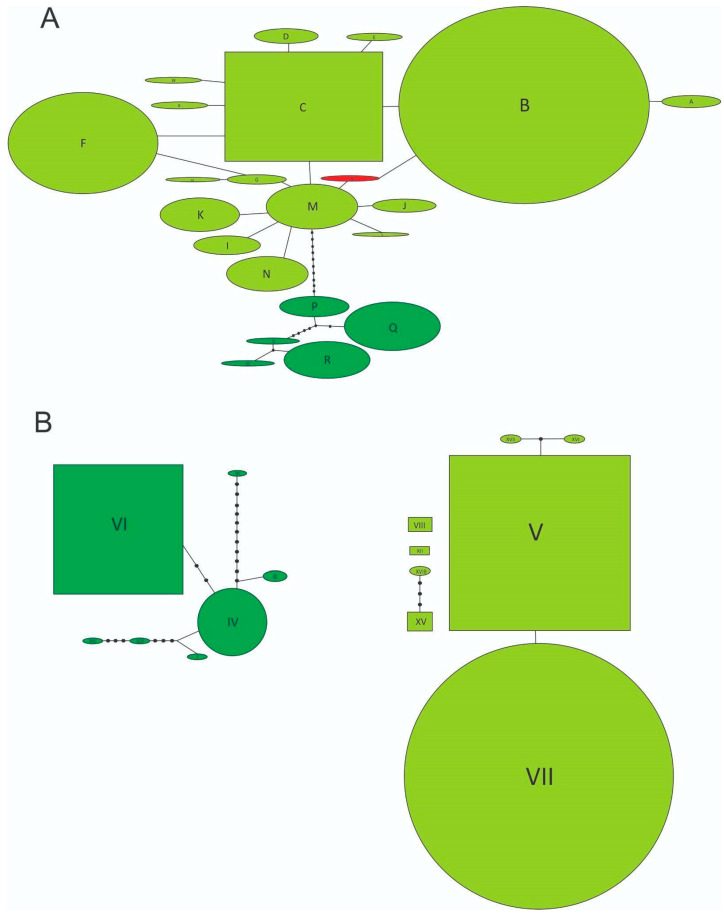
(**A**) Statistical parsimony network of mitochondrial haplotypes. Lines represent the most-parsimonious relationships between haplotypes and indicate one mutational step between two haplotypes. Dark green open circles represent individual haplotypes from the Forest Clade, light green open circles represent individual haplotypes from the Grassland Clade, red open circle represents the novel Y haplotype, and unlabeled black circles indicate inferred intermediate haplotypes not found in the samples. Rectangles indicate possible ancestral haplotypes. Circle size is proportional to haplotype frequency. (**B**) Statistical parsimony network of nuclear alleles and the graphical representation follows nomenclature explained in (**A**). Individual haplotype and allele codes follow Table 1A.

By studying a sample of 71 individuals coming from 13 locations from three southern states from the US, namely California, Georgia, and Florida, we found only two multilocus genotypes. While most of the specimens showed the B-V genotype (COI-ITS1), only two individuals from Tulare Co., California (3%), had the Y-V combination (Table 1, Figure 1A), with Y being a closely related and novel haplotype for *N. cervinus*. Statistical parsimony networks show that both components of the multilocus genotype belong to the Grassland clade (Figure 2A,B) and that the novel Y mitochondrial sequence is, indeed, derived from B by a single synonymous mutation replacing a G with an A in a third codon position (Figure 2A, Accession number: ON682730). Two scenarios can be posed about the origin of this new variant: either it already occurred within the native range and was independently introduced to California or it was recently derived from B or M exclusively at this location.

Even though no new genotype combinations were found within the native area, some new geographic distribution details of known multilocus genotypes were unveiled. In the new samples from the Yungas Montana jungle (AMA, SSJ), we found two different combinations, B-V and C-VI, extending the range of these genotypes northwards (Figure 1B). The C-VI variant was also retrieved from the new sample collected from the Atlantic coast (PIM). Additionally, this survey also showed that the M-VII combination, a genotype typical from the riverbanks of the Paraná river, is also present in Buenos Aires City (BA sample). This increases the available variation in the surroundings of the Buenos Aires port, the origin of most of the maritime commercial trade worldwide, and therefore, the putative source of most *N. cervinus* introductions from South America.

Table 2 shows that mitochondrial and nuclear genetic variation estimations, based on both the *Pi* and *k* measures in the native range, surpass those from the introduced range by one order of magnitude. Within the introduced range, most of the COI variation is explained by the localities from Chile (INSA locality group), which harbor distinct COI haplotypes (Table 1). Thus, the overall genetic differentiation estimates for all introduced locality groups only yield significant indexes (Kst = 0.20437 *) when computed including INSA, since no variation is found in the other localities worldwide because all others carry mitochondrial haplotype B. Similarly, most of the ITS1 allelic variation in the introduced range is also explained by the INSA samples, which carry three different alleles (V, VII, and XVIII). All other introduced populations carry alleles V and/or VII, which differ by only one nucleotide insertion/deletion event within a polyA region. In summary, all introduced areas (except for INSA) display one of three multilocus genotypes: either B-V, Y-V, or B-VII, a result that contrasts with the wide variety of multilocus genotypes that can be found within the native region (up to 32 genotypes, excluding ITS1 hybrid genotypes, as listed in Table 1). This can explain the ten-fold decrease in variation within the introduced range.

Comparison of all the introduced samples investigated in the present work, with the probability distribution map based on ecological niche modeling obtained by [19], shows that these weevils are, indeed, expanding in areas modeled as ecologically unfavorable (see Figure 6 in [19]). 

## 4. Discussion

As seen in previous works, in spite of the high levels of genetic variation displayed by this weevil within its native range (Figure 1B), only the carriers of three highly related multilocus genotypes, B-V, B-VII, and the newly derived Y-V-, successfully colonized almost all sampled localities around the world [19,20], where this pest insect is believed to be introduced by commercial trade. Particularly, the B-V combination recorded herein across the southern US was also found in the insular US state of Hawaii and other islands from Oceania, such as Australia and New Zealand, and in low frequency in central and northeastern Argentina (Table 1, Figure 1B). Thus, the most likely scenario of expansion of this particular variant, that is less frequent than the B-VII genotype in the area of origin, would propose that B-V expanded through the continental US, possibly as a byproduct of the traffic between different US states. Because records of *N. cervinus* in the US predate those of other areas where B-V occurs (e.g., Australia) [47], it appears more likely that the US could be a secondary source of introductions into those areas [32,48]. Even though they appear less likely, repeated introductions from the Argentine plains cannot be completely ruled out; including more molecular markers in future studies might help disentangle these routes of dispersion and colonization. Although higher levels of mitochondrial diversity were found in a small portion of the introduced range, the INSA locality group may have a peculiar introduction history, being the product of human mediated expansion by land (rather than commercial transport through the port of Buenos Aires). Otherwise, most of the invaded area depicts low levels of genetic variation.

Surveys of genetic variation in additional countries where *N. cervinus* has established successfully contribute to demonstrate that an invasive genotype is able to cope with a diversity of environmental conditions, similar to those present in such different areas as California and Florida. Certain traits, such as habitat tolerance or the ability to cope with dryer and cooler conditions than those prevailing in the forests where this species originated [19], might be seen as pre-adaptations, allowing for establishment in the southern US. The fact that a multilocus genotype not found in previous surveys was also found in the state of California does not refute our hypothesis of a successful invader genotype, since the mitochondrial haplotype Y is very likely a derivative of B. Two scenarios can be posed regarding the origin of this new variant: either it already evolved within the native range where it occurs in low frequency, was introduced to California along with other variants and then became more frequent through genetic drift or natural selection, or it was recently derived from B exclusively at this location. Considering the high substitution rate of the insect mitochondrial DNA [49], the derived position of this haplotype in the network, its extremely low frequency in a single location out of 70 and the fact that exhaustive sampling of the native range revealed occurrence of other haplotypes in lower frequency than Y, we lean towards the idea of an in-situ origin, rather than multiple introductions.

Comparison of the present survey with the ecological niche modeling analysis previously performed by [19] suggests that invaded areas in the continental US are non-suitable for *N. cervinus* establishment (continental US locations fell in areas where the scale color bar in Figure 6 of [19] shows this species is outside the range of the predicted favorable environmental conditions). Thus, it could be proposed that carriers of the B-V genotype and its derivatives Y-V and B-VII perform well in novel conditions for this weevil. The reasons behind the apparent success beyond the area of suitability are hard to disentangle and could be the result of a combination of conditions and factors that we explore below, namely the inherent advantages of parthenogenetic reproduction and the resulting lack of recombination, as well as the lack of biotic interactions that regulate population size in the native area [50]. On one hand, failures in mate-finding during the process of biological invasion [51] can be overcome by parthenogenetic reproduction, thus facilitating the successful colonization of new areas by a single individual. Furthermore, many invading species spread through stratified dispersal, in which new colonies arrive and establish beyond the infested area, grow, and eventually coalesce with the expanding population front; the result is an accelerated spread rate, relative to diffusive spread [52]. Consequently, the formation of new colonies can have a profound impact on the speed of invasion [53,54,55]. The fact that a single *N. cervinus* female might initialize a new population without invoking males has immediate implications to its invasion dynamics, including the rate of spread, as it was already observed in other insects, such as the hemlock woolly adelgid *Adelges tsugae* [56]. Another component of establishment success could be the linkage disequilibrium between genomic variants as a byproduct of parthenogenetic reproduction detected by [24], originating co-adapted gene complexes or supergenes, i.e., clusters of tightly linked loci [57]. Lack of natural enemies, such as parasitoids [58,59], may also be beneficial for establishment in new areas, where population size may increase without biotic restrictions. 

Genetically homogeneous US *N. cervinus* populations could be the product of one or multiple introduction pulses carrying the most prevalent invasive (or expanding) multilocus genotype B-V. Alternatively, the widespread distribution of the B-V multilocus genotype in the introduced range could be the product of multiple introduction pulses from genetically diverse sources, with several *N. cervinus* genotypes entering with the same probability. Should this be the case, the result is that only the B-V (and its derivative Y-V) was able to successfully establish and expand its range across North America. In either scenario, parthenogenetic reproduction could aid establishment by impeding recombination. In addition, parthenogenesis confers demographic advantages by avoiding the two-fold cost of sex [60], thus doubling the rate of population growth [61]. Only a few parthenogenetic weevil species have established populations far from their areas of origin in suitable or moderately suitable environments [16,62,63], and *N. cervinus* is the only worldwide distributed species that thrives in a diversity of habitats. Thus, the remarkably high frequency of the B-V and the B-VII genotypes is intriguing and prompts initiating future studies in search of possible selective effects favoring the geographic range expansion in *N. cervinus* into a diversity of habitats modeled to have low to null probabilities of occurrence [19,20].

At first sight, the genetic variation of *N. cervinus* in introduced areas is fairly low to null, as the genetic divergence analysis indicated. Strikingly, [31] showed that *N. cervinus* is capable of modulating gene expression responses to different types of stressful situations, such as taxing host plants. In the absence of genetic variation, epigenetic regulation may be an important mechanism of successful colonization of novel, adverse areas. It is puzzling that another parthenogenetic species, the whitefringed weevil *Naupactus leucoloma*, with the same evolutionarily favorable core shared gene expression regime for responding to different types of stressful situations as *N. cervinus* [31], has only established introduced populations in suitable areas [16], possibly signaling that the Fuller’s rose weevil is a superior colonizer. Is it possible then that *N. cervinus* may have another source of adaptive evolution, in addition to expression plasticity? Is it possible that together with epigenetic variation compensating for decreased genetic variation, the success of the Fuller’s rose weevil may also have a genetic basis?

It seems that *N. cervinus* adjusts to the “general purpose genotype (GPG) hypothesis” [64]. It predicts that selection on multiple clonal lineages will favor ones that show an enhanced plasticity in abiotic varying habitats [65,66]. Other parthenogenetic weevils, such as the black vine weevil *Otiorhynchus sulcatus* [67], were proposed as having a GPG. In the case of *N. cervinus*, we propose that the genetic combinations B-V/B-VII/Y-V could be closely related variants of a GPG lineage. Understanding the mechanisms underlying biological invasions and successful establishment to novel environments remains a fundamental challenge, particularly in small populations lacking genetic variation, such as those in the present work. We are currently performing a genome scan of adaptive loci and designing experiments in common gardens to compare native vs. invasive populations of *N. cervinus* to identify candidate genes and characters involved in the colonization ability of marginal areas in this weevil. Then, we will be able to better understand the potentially adaptive basis of the geographic and expansion process of this successful invader.

## Figures and Tables

**Table 1:** Locality and multilocus genotype information for all native and introduced sampling locations for *Naupactus cervinus*. Locality groups are clusters of geographically close localities in the introduced range presumed to be the result of single introduction events: INUSCO1 and 2: Localities from Continental US area 1 (Florida and Georgia) and 2 (California); INUSI: US Insular localities from Hawaii; INSA: Localities from South America; INEU: Localities from Europe; INAUN: Localities from Australia and New Zealand and INPI: Localities from the Pacific Islands, French Polynesia. Individual locality codes are used as labels in Figure 1, and those followed by an asterisk (*) are newly included in this study. N: indicates the number of sequences for each gene region from that locality. Combined genotype codes list COI and ITS1 variants in that order, all ITS1 genotypes are roman numerals with the exception of four heterozygote genotypes labeled H1-4. Heterozygote genotypes are 1: VI-VIII, 2: I-XIX, 3: XIII-XII, and 4: VI-XVIII. When localities harbor different multilocus genotypes, numbers in parentheses beside genotype designations indicate the number of weevils that carry each genotype in that locality. The table footnote describes superscript letters that accompany each multilocus genotype indicating the origin of those sequences (obtained for this study or from previous studies).

Locality Group	Locality Details	Code	Coordinates (DDM)	N (CO1/ITS1)	Multilocus Genotype
Native range (40)					
	AR-Bs. As., Benavídez	BE	34°24′ S; 58°41′ W	7/7	C-H_1_(6), F-VI(1)b
	AR-Bs. As., Buenos Aires	BA	34°36′ S; 58°26′ W	16/16	B-V(3) ^a^,C-VI(1) ^b^,F-VI(5) ^a^, G-VI(1) ^b^,G-VII(1) ^b^, H-VI(1) ^a^, M-VII (4) ^e^
	AR-Bs. As., Cardales	CA	34°18′ S; 58°57′ W	5/5	B-VII(3) ^a^,G-VI(2) ^a^
	AR-Bs. As., Reserva Otamendi	RO	34°14′ S; 58°52′ W	11/11	N-VII ^a^
	AR-Bs. As., Parque Pereyra Iraola	PI	34°50′ S; 58°8′ W	12/12	B-VII(1) ^a^, D-VII(8) ^a^, F-VI(3) ^a^
	AR- Bs. As., Pergamino	PE	33°54′ S; 60°35′ W	1/1	B-VII ^a^
	AR-Bs. As., Talavera Island	TI	34°10′ S; 58°30′ W	17/17	B-VII(1) ^a^, F-VI(6) ^a^, K-VII(1) ^a^, M-VII (9) ^a^
	AR-Bs. As., Tandil	TA	37°19′ S; 59°08′ W	7/7	B-V(5) ^a^, F-VI(2) ^a^
	AR-Bs. As., Tres Lomas	TL	36°28′ S; 62°52′ W	4/4	B-VII ^a^
	AR-Bs. As., Zárate	ZA	34°06′ S; 59°01′ W	15/15	B-VII(1) ^a^, K-VII(14) ^a^
	AR-Bs. As., Pinamar	PIM*	37°6.57′ S; 56°52.69′ W	1/1	C-VI ^d^
	AR-Córdoba, La Falda	LF	31°05′ S; 64°29′ W	7/7	B-VII ^a^
	AR-Córdoba, Río Cuarto	RC	33°08′ S; 64°21′ W	5/5	A-VII(3) ^a^, B-VII(2) ^a^
	AR-Corrientes, Yapeyú	YA	29°28′ S; 56°50′ W	5/5	C-H_1_(4) ^a^, E-H_1_(1) ^a^
	AR-E. Ríos, Brazo Largo	BL	33°54′ S, 58°53′ W	11/11	M-VII(7) ^a^, C-H_4_(4) ^a^
	AR-E. Ríos, Cerrito	CE	31°34′ S, 60°03′ W	3/3	C-XVI(1) ^b^, W-V(2) ^b^
	AR-E. Ríos, Chajarí	CHJ	30°47′ S, 57°59′ W	6/6	F-VII(1) ^a^, F-VI(5) ^a^
	AR-E. Ríos, El Palmar	EP	31°50′ S, 58°17′ W	5/5	F-VI ^a^
	AR-E. Ríos, Gualeguaychú	GU	33°01′ S, 58°31′ W	16/16	F-VI(11) ^a^, M-VII(5) ^a^
	AR-E. Ríos, La Paz	LP	30°45′ S, 59°38′ W	6/6	R-H_2b_
	AR-E. Ríos, Salto Grande	SG	31°23′ S, 58°01′ W	4/4	F-VI ^a^
	AR-E. Ríos, Santa Elena	SE	30°56′ S, 59°48′ W	2/2	X-XVII ^b^
	AR-Mendoza, Godoy Cruz	GC	32°56′ S, 68°50′ W	2/2	A-VII ^a^
	AR-Mendoza, Mendoza	ME	33°30′ S, 69°W	3/3	BVII ^a^
	AR-Misiones, Cerro Azul	CA	27°38′ S, 55°30′ W	12/12	QIV(9) ^a^, TX(2) ^c^, SIX(1) ^c^
	AR-Misiones, Oberá	OB	27°29′ S, 55°08′ W	1/1	QIII ^a^
	AR-Tucumán, San Miguel de Tucumán	TU	26°46′ S, 65°13′ W	1/1	AV ^b^
	AR-Tucuman, Amaicha	AMA *	26°35.74′ S; 65°55.33′ W	1/1	BV ^e^
	AR-Jujuy, San Salvador de Jujuy	SSJ *	24°10.82′ S; 65°18.53′ W	1/1	CVI ^e^
	BR-PR, Laranjeiras do Sul, LS	LS	25°24′ S, 52°24′ W	11/11	VH_3_(8) ^b^, VXII(1) ^b^, VXIV(1) ^b^, VXIII(1) ^b^
	BR-PR, Ponta Grossa, PG	PG	25°05′ S, 50°09′ W	14/14	CH_4_(5) ^b^, RI(1) ^a^, FVI(4) ^b^, RH_2_(4) ^a^
	BR-PR, Toledo, TO	TO	24°42′ S, 53°44′ W	10/10	CH_1_(6) ^a^, QIV(4) ^b^
	BR-RG do Sul, Alegrete	AL	29°46′ S, 55°47′ W	6/6	CH_1_(1) ^a^, FVI(4) ^b^, CVIII(1) ^b^
	BR-RG do Sul, Bozano	BO	28°35′ S, 53°59′ W	4/4	QII(3) ^a^, CII(1) ^a^
	BR-RG do Sul, Ijui	IJ	28°23′ S, 53°54′ W	9/9	RH_2_(2) ^b^, OXV(1) ^b^, CXV(2) ^b^, CH(4) ^a^
	BR-RG do Sul, Itaára	IT	29°36 S, 53°45 W	7/7	CH_1_(4) ^a^, CVIII(3) ^b^
	BR-RG do Sul, Jari	JA	29°17′ S, 54°13′ W	17/17	CXV(5) ^b^, CH_1_(12) ^a^
	BR-RG do Sul, Santa Maria	SM	29°40′ S, 53°47′ W	14/14	QIV(11) ^a^, CH_1_(3) ^b^
	BR-RG do Sul, São Sepé	SS	30°10′ S, 53°34′ W	6/6	PIV ^a^
	BR-SC, Chapecó	CHP	27°03′ S, 52°36′ W	8/8	UXI(7) ^c^, EH_1_(1) ^a^
Introduced range (30)					
INUSCO1	US-GA, USDA station, Byron	BYR-68 *	32°39.22′ N; 083°42.91′ W	4/3	4/3
	US-GA, KOA campground, Forsyth	FOR-67 *	33°02.27′ N; 083°55.55′ W	3/3	B-V ^e^
	US-GA, Howard North Lake	HOW-69 *	32°32.61′ N; 083°44.34′ W	5/7	B-V ^e^
	US-GA, Post Street Park, Douglasville	POST-70 *	33°42.39′ N; 084°50.56′ W	5/5	B-V ^e^
	US-FL, Oleno State Park, High Springs	OLEA-72 *	29°49.44′ N; 082°35.39′ W	2/2	B-V ^e^
	US-FL, Quincy Research Station	QUIN-71 *	30°32.71′ N; 84°35.75′ W	4/7	B-V ^e^
INUSCO2	US-CA, Rt. 220, Tulare Co.	TULONE *	36°21.11′ N; 119°04.84′ W	9/9	B-V ^e^
	US-CA, 7 km. from Lindcove Station, Tulare Co.	TULTWO *	36°19.99′ N; 119°05.60′ W	7/8	B-V(5) ^e^, Y-V(2) ^e^
	US-CA, Lindcove Station, Tulare Co.	TULTHREE *	36°21.22′ N; 119°83.38′ W	4/5	B-V ^e^
	US-CA, Lally farms, Av. 124 & Rd. 232,Tulare Co.	TULFOUR *	36°1.39′ N; 119°4.56′ W	3/7	B-V ^e^
	US-CA, Brekenridge Rd. and Pepper Dr., Kern Co.	KERONE *	35°21.63′ N; 118°52.44′ W	4/4	B-V ^e^
	US-CA, Pepper Ranch, Kern Co.	KERTWO *	35°21.50′ N; 118°51.80′ W	4/6	B-V ^e^
	US-CA, Valpredo ranch, Kern Co.	KERFOUR *	36°0.97′ N; 119°3.13′ W	2/4	B-V ^e^
INUSI	US-PI, Hawaii, Big Island	BI	19°36′ N; 155°39′ W	1/1	B-V ^b^
	US-PI, Hawaii, Kauai	KA	22°07′ N; 159°31′ W	2/2	B-V ^b^
	US-PI, Hawaii, Maui	MU	20°50′ N; 156°20′ W	1/1	B-V ^b^
	US-PI, Hawaii, Oahu	OH	21°28′ N; 157°59′ W	1/1	B-V ^d^
INSA	CHI/PI, Isla de Pascua (Rapa Nui),	IP/EL	27°08′ S; 109°26′ W	7/7	I-VII ^a^
	CHI, Bio Bio, Chillan,	CHI	36°36′ S; 72°06′ W	8/8	B-VII ^d^
	CHI, Santiago	SC	33°26′ S; 70°29′ W	6/6	B-VII ^a^
	CHI, Vallenar	VAR	28°57′ S; 71°15′ W	11/11	I-VII(4) ^a^, J-VII(7) ^a^
	CHI, La Serena	LSE	29°50′ S; 71°14′ W	3/3	M-VII(2) ^b^, R-XVIII(1) ^b^
INEU	ES, Canary Islands, Tenerife	TE	27°27′ N; 16°14′ W	5/5	B-VII ^a^
	ES, Valencia	VAL	39°29′ N; 00°23′ W	10/10	B-VII ^a^
INAUN	AU, Victoria, Vermont	VE	37°50′ S; 145°11′ E	1/1	B-V ^d^
	AU, Victoria, Tatura	TA	36°26′S; 145°13′ E	1/1	B-V ^d^
	NZ, Auckland, Awhitu	AW	37°05′ S; 174°39′ E	1/1	B-V ^d^
	NZ, Bay of Plenty, Matapihi	MA	37°41′ S; 176°11′ E	1/1	B-V ^d^
INPI	PI, French Polynesia, Rapa Island (Rapa Iti)	RI	27°32′ S; 144°20′ W	1/1	B-VII ^a^
	PI, French Polynesia, Tahiti	TH	17°52′ S, 149°56′ W	3/3	B-VII ^a^

a: Extracted from [24]; b: Extracted from [19]; c: Extracted from [34]; d: Extracted from [35]; e: Obtained for the present study.

**Table 2:** Genetic diversity comparisons within the introduced and native areas and for the total dataset for both gene regions. Calculations include the average number of nucleotide differences: *k* and nucleotide diversity, *Pi*.

Area	Sequenced Region/Number of Sequences	Within Areas		
		Number of Polymorphic Sites/Mutations	k	Pi
Native	CO1/ 365	17/17	2.850	0.01183
	ITS1/ 287	27/30	6.301	0.01011
Introduced	CO1/ 114	7/7	0.281	0.00117
	ITS1/ 114	9/9	0.158	0.00025
Total	CO1/479	18/18	2.343	0.00972
	ITS1/ 401	27/30	5.586	0.00897

## Data Availability

The GenBank Accession Numbers of the final dataset can be found in the body of the manuscript and in Table S1.

## Supplementary Materials

The following supporting information can be downloaded at: https://www.mdpi.com/article/10.3390/insects14020113/s1, Table S1: GenBank Accession Numbers of COI haplotypes and ITS1 alleles of *Naupactus cervinus*.
