# Supplementary material for: Genetically Depauperate and Still Successful: Few Multilocus Genotypes of the Introduced Parthenogenetic Weevil Naupactus cervinus (Coleoptera: Curculionidae) Prevail in the Continental United States
Source: Insects. 2023 Jan 22;14(2):113. doi: 10.3390/insects14020113 (PMC9958569; doi:10.3390/insects14020113)
Supplement: Supplementary file 1 [file insects-14-00113-s001.zip › insects-2140009-supplementary.pdf]

Table S1 GenBank Accession Numbers for mitochondrial haplotypes and nuclear alleles retrieved from Rodriguero et al. 2010, 2013, 2016, 2018.

COI Haplotypes	Accession Numbers
A	GQ406827.1
B	GQ406828.1
C	GQ406829.1
D	GQ406830.1
E	GQ406831.1
F	GQ406832.1
G	GQ406833.1
H	GQ406834.1
I	GQ406835.1
J	GQ406836.1
K	GQ406837.1
L	GQ406838.1
M	GQ406839.1
N	GQ406840.1
O	KX074095.1
P	GQ406841.1
Q	GQ406842.1
R	GQ406843.1
S	JX440490.1
T	KC614562.1
U	GU727685.2
V	KX074096.1
W	KX074097.1
X	KX074098.1

ITS1 Alleles	Accession Numbers
I	GQ406818.1
II	GQ406819.1
III	GQ406820.1
IV	GQ406821.1
V	GQ406822.1
VI	GQ406824.1
VII	GQ406823.1
VIII	GQ406825.1
IX	JX440499.1
X	KC614561.1
XI	JX440500.1
XII	KX074088.1
XIII	KX074089.1
XIV	KX074090.1
XV	KX074091.1
XVI	KX074092.1
XVII	KX074093.1
XVIII	KX074094.1
XIX	KY305942.1
